# ​Aging-related gene signatures as potential biomarkers in ischemic stroke: an integrated bioinformatics and machine learning study

**DOI:** 10.1186/s12883-026-04692-0

**Published:** 2026-02-17

**Authors:** Peilu Wang, Yan Wang, Dongliang Wang, Yingxuan Li, Zhenyu Yin, Fuqing Zhang

**Affiliations:** 1 Department of Neurology, Second Hospital of Tianjin Medical University, Tianjin, 300211 China; 2 Department of Neurology, Tianjin Dongli Hospital, Tianjin, 300300 China; 3 Department of Geriatrics, Tianjin Medical University General Hospital, Tianjin, 300052 China; 4 Department of General Practice, Tianjin Medical University General Hospital, Tianjin, 300052 China

**Keywords:** Ischemic stroke, Aging, Biomarkers, Immune infiltration, Machine learning, Molecular docking

## Abstract

**​Background:**

Ischemic stroke (IS) and aging share similar pathophysiological features, including vascular dysfunction, inflammatory responses, and oxidative stress. However, the underlying molecular mechanisms remain unclear. This study aimed to identify key shared drive genes between IS and aging through integrated multi-omics analysis and machine learning approaches, and to explore their diagnostic and therapeutic potential.

**​Methods:**

Based on the GSE22255 and GSE58294 datasets, differentially expressed genes (DEGs) associated with IS were screened using differential expression analysis and weighted gene co-expression network analysis (WGCNA). The overlapping genes between IS-related DEGs and aging-related genes (ARGs) were identified as aging-related DEGs (ARDEGs). Further refinement of core genes was performed through Gene Ontology (GO)/Kyoto Encyclopedia of Genes and Genomes (KEGG) enrichment analysis, protein-protein interaction (PPI) network construction, and machine learning algorithms (LASSO regression and support vector machine [SVM]). Immune cell infiltration was evaluated using CIBERSORT, and gene expression patterns were validated via the DISCO single-cell database. Finally, molecular docking and drug database screening were employed to predict potential therapeutic targets.

**​Results:**

A total of 279 IS-related DEGs were identified, among which 29 showed significant overlap with ARGs (ARDEGs). WGCNA revealed that the MEcyan module exhibited a strong negative correlation with IS phenotypes (r = -0.63). Machine learning algorithms identified JUP, UQCRC1, and MRPL41 as core potential diagnostic biomarkers, with area under the curve (AUC) values all exceeding 0.7. Immune infiltration analysis demonstrated a significant increase in M1 macrophages and neutrophils, along with a reduction in CD8+ T cells in IS patients. Single-cell data confirmed the specific expression of these core genes in neurons and immune cells. Molecular docking suggested that the herbicide atrazine may target these genes (binding energy < -5.7 kcal/mol), while hsa-miR-30c-5p was predicted to regulate JUP and UQCRC1.

**​Conclusion:**

This study elucidates key shared genes and immune microenvironment features between IS and aging, proposing JUP, UQCRC1, and MRPL41 as potential diagnostic biomarkers. Furthermore, atrazine was identified in silicoas a potential interacting molecule, suggesting the druggability of these target proteins​ and providing a starting point for future drug discovery efforts in IS.

**Supplementary Information:**

The online version contains supplementary material available at 10.1186/s12883-026-04692-0.

## Introduction

Ischemic stroke (IS), accounting for 80% of all stroke cases, represents the second leading cause of death among non-communicable diseases and ranks third in global disability burden [1, 2]. The ischemic cascade involves multifaceted cellular damage to neurons, glia, and vasculature, accompanied by infiltration of lymphocytes, granulocytes, and rare macrophage subsets that trigger neuroinflammatory responses [3]. Despite this understanding, the pathological complexity and heterogeneity of IS remain incompletely elucidated, with therapeutic options severely limited. Currently, tissue plasminogen activator (tPA) stands as the sole FDA-approved thrombolytic agent, yet its narrow 4.5-hour treatment window post-symptom onset underscores the urgent need for novel potential diagnostic biomarkers and evidence-based therapies [4].

Chronological age constitutes the most significant non-modifiable risk factor for IS, correlating with increased susceptibility and poorer outcomes [5, 6]. The intersection of aging and IS pathophysiology manifests through shared features including atherosclerosis, hypertension, metabolic dysfunction, and notably, chronic low-grade inflammation [7, 8]. Emerging evidence highlights that biological aging metrics - encompassing clinical biomarkers [9], leukocyte telomere length [10], and DNA methylation patterns [11] - serve as robust predictors of stroke incidence. A prospective cohort study of 7,396 IS/TIA patients further established that accelerated biological age positively associates with both short- and long-term adverse outcomes [12]. Crucially, CNS-associated macrophages (CAMs) have been identified as pivotal regulators of neuroimmune responses during aging, modulating post-stroke immunological cascades [13], thereby implicating immunosenescence as a mechanistic link between aging and IS.

**Fig. 1:**
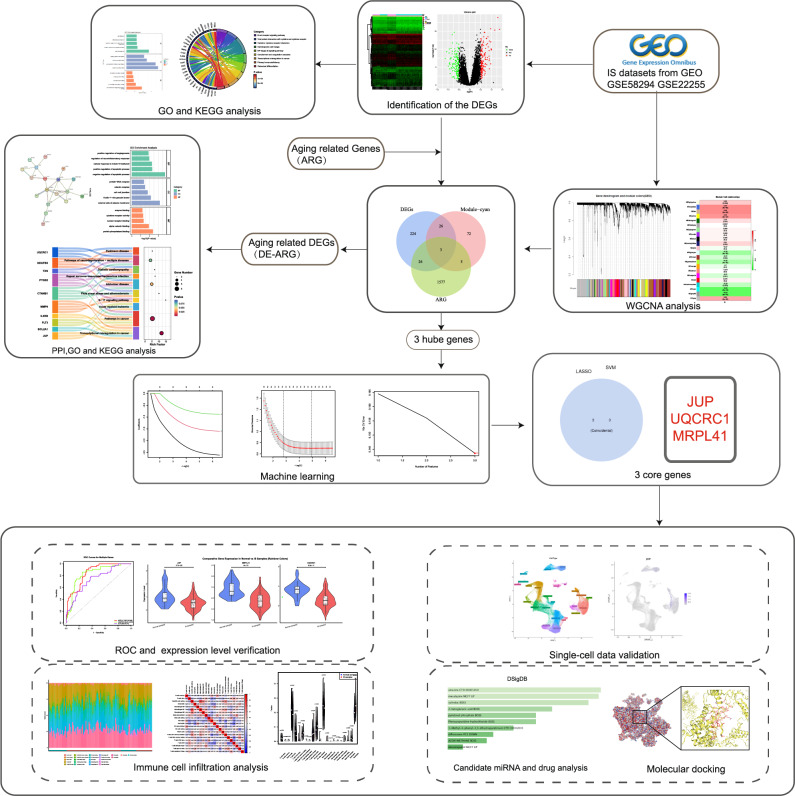
Research flowchart

The shared pathophysiological axis of IS and aging involves vascular dysfunction, enhanced oxidative stress, and neurovascular unit impairment. While clinical correlations are well-documented, the genetic and molecular underpinnings remain largely unexplored. Transcriptomics coupled with machine learning has emerged as a powerful paradigm for unraveling disease mechanisms, identifying biomarker signatures, and discovering therapeutic targets. This study leverages cross-disease genomic integration to: (i) delineate shared gene networks between IS and aging via weighted gene co-expression network analysis (WGCNA); (ii) characterize enriched biological pathways through integrative GO/KEGG analysis; (iii) identify diagnostic hub genes using LASSO regression and SVM-RFE algorithms; (iv) evaluate immune cell infiltration patterns across molecular subtypes; and (v) predict potential therapeutic agents via molecular docking approaches. Figure 1 showed the workflow chart of the present study.

## Methods

### Transcriptomic data preprocessing

Transcriptomic datasets for Ischemic stroke (IS) were retrieved from the GEO database. The IS cohorts comprised 20 IS patients and 20 healthy controls (HCs) from GSE22255, plus 69 IS patients and 23 HCs from GSE58294(Table 1). Gene symbols were mapped to the expression data, with median values assigned in cases of multiple matches. Data normalization was performed using the log2(X + 1) transformation. A merged gene expression matrix containing overlapping genes across all datasets was subsequently generated. We applied the ComBat algorithm (from the sva R package) to correct for batch effects in the merged training set (GSE22255 and GSE58294).

**Table 1:** Basic information of GEO datasets used in the study

ID	GSE series	Samples	Source types	Platform	Group
1	GSE22255	20 IS patients and 20 normal controls	PBMCs	GPL570	Discovery cohort
2	GSE58294	69 IS patients and 23 normal controls	whole blood	GPL570	Discovery cohort
3	GSE16561	39 IS patients and 24 normal controls	whole blood	GPL6883	Validation cohort

*IS* Ischemic stroke, *PBMCs *Peripheral blood mononuclear cells

### Diagnostic biomarker initial screening

Differentially expressed genes (DEGs) were identified between IS patients and HCs within the combined dataset using the limma package (a tool for differential expression analysis of RNA-seq and microarray data). Genes with an adjusted *P*-value (P.adj) < 0.05 and an absolute Log Fold Change (|LogFC|) > 0.5 were selected as significant DEGs. *P*-values were corrected for multiple testing using the Benjamini-Hochberg method. Weighted Gene Co-expression Network Analysis (WGCNA) (24) was applied to the combined IS dataset using all genes to construct an input matrix. A soft-thresholding power ranging from 1 to 20 was assessed, and the optimal power was selected to achieve a scale-free topology. The adjacency matrix was transformed into a topological overlap matrix (TOM). Hierarchical clustering with average linkage was performed based on TOM to define gene modules, setting a minimum module size of 100 genes. Similar modules were subsequently merged. Pearson correlation coefficients were calculated to assess module-trait relationships (disease phenotype). The modules demonstrating the strongest positive and negative correlations with the disease phenotype were designated as key modules. Gene significance (GS) quantified the association strength between individual genes and the trait. Module membership (MM) represented the correlation between a gene’s expression profile and the module eigengene.

To identify aging-related genes, the GeneCards database was queried using the keyword “aging“ [14]. Following their retrieval, the gene identifiers were converted in R with the homologene package for subsequent analysis. Further information on these aging-related genes is available in Table S1.

### GO and KEGG enrichment analysis

Enrichment analyses for Gene Ontology (GO) and Kyoto Encyclopedia of Genes and Genomes (KEGG) pathways were performed on the candidate shared driver genes using the cluster Profiler package (a tool for comparing and visualizing biological themes among gene clusters). GO terms encompass Biological Processes (BP), Molecular Functions (MF), and Cellular Components (CC). KEGG analysis provided pathway annotations. Terms with *P* < 0.05 were considered significantly enriched.

### PPI network construction

The 29 AR-DEGs were submitted to the STRING database (https://string-db.org/) to evaluate protein-protein interaction (PPI) relationships and remove redundant genes.

### Machine learning-based diagnostic biomarker screening​

Three core genes, identified from the intersection of aging-related differentially expressed genes (ARDEGs) and the Weighted Gene Co-expression Network Analysis (WGCNA), were used as input features for the machine learning models. Two machine learning algorithms, Support Vector Machine (SVM) and Least Absolute Shrinkage and Selection Operator (LASSO) regression, were employed using the training dataset (GSE22255 and GSE58294) for variable selection and model development within a tenfold cross-validation framework [15].

### ​Immune infiltration analysis

The relative proportions of 22 immune cell subpopulations within the IS cohorts were estimated using the CIBERSORT algorithm based on immune-related gene expression profiles. *p* < 0.05 was used to filter the samples.An immune cell composition matrix was generated by integrating these estimates. The Spearman’s rank correlation coefficient was then calculated to assess the association between the core biomarkers identified in Sect. 2.5 and the infiltration levels of specific immune cell types. The resulting *P*-values were adjusted for multiple comparisons using the Benjamini-Hochberg method.

### Validation at single-cell transcriptome resolution

Expression levels of key genes were validated across healthy cell populations using the DISCO database (https://www.immunesinglecell.org/). DISCO is a deeply integrated single-cell multi-omics platform, currently aggregating data from over 18 million cells derived from 4,593 samples. This comprehensive resource covers 107 distinct tissues/cell lines/organoids, 158 disease types, and 20 technology platforms. As a multifunctional tool, DISCO not only facilitates the querying of published single-cell datasets but also enables efficient integrative analysis incorporating user-submitted data. These detailed cellular atlases provide invaluable reference maps for studying healthy versus diseased cells.

### Identification of MiRNA and drug candidates

To identify potential miRNA and drug candidates targeting common pathology in Ischemic stroke , we utilized the Drug Signature Database (DSigDB) via the Enrichr web platform (https://amp.pharm.mssm.edu/Enrichr/).

### Molecular docking

The three-dimensional structures of target proteins were obtained in PDB format from the RCSB Protein Data Bank (https://www.rcsb.org/) *JUP* ( PDB ID-3IFQ), *UQCRC1*(PDB ID-5XTE), *MRPL41* (PDB ID-7NQL)), while molecular structures of *atrazine* (CID 2256) were acquired from PubChem database. Protein-ligand interactions were analyzed through molecular docking simulations performed on the CB-Dock2 platform (https://cadd.labshare.cn/cb-dock2/). Binding affinities were calculated, with negative values (< 0 kcal/mol) indicating thermodynamically favorable binding and values <-5 kcal/mol representing stable interactions with potential biological significance.

## Results

### Identification of shared DEGs and WGCNA analysis in IS datasets

**Fig. 2:**
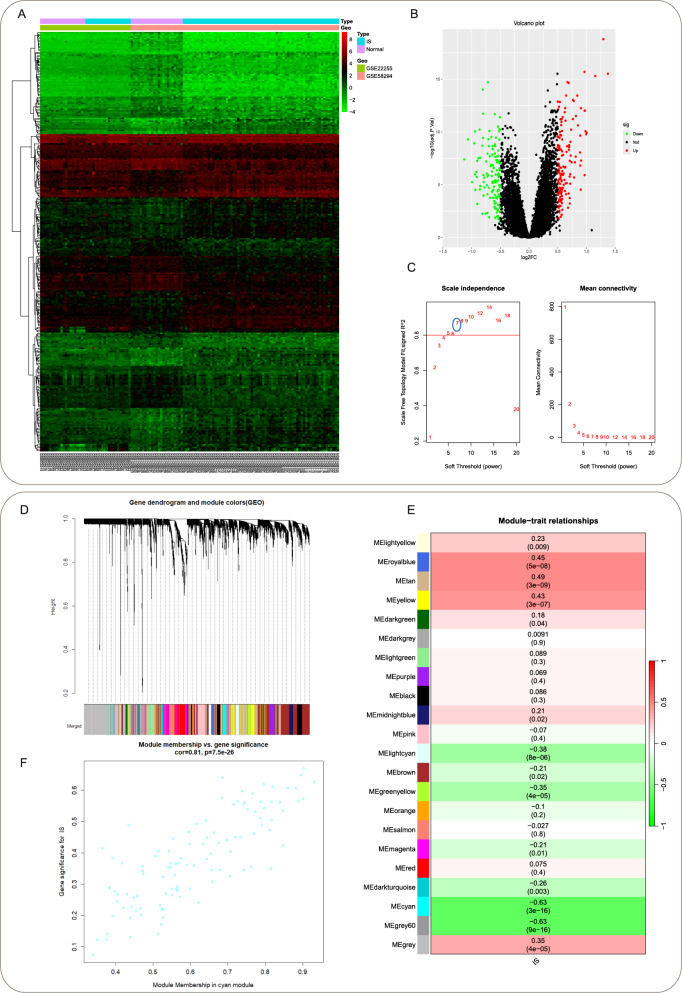
Identification of shared differentially expressed genes (DEGs) in IS datasets and WGCNA analysis. ​**A** Heatmap of differential gene expression in IS datasets. ​**B** Volcano plot illustrating the distribution of DEGs in IS, with downregulated genes in green and upregulated genes in red. ​**C**, **D** Scale independence, soft thresholding (β)-dependent mean connectivity, and gene clustering dendrogram in the IS cohort. ​**E**, **F** Heatmap depicting the correlation between module eigengenes and clinical phenotypes in IS samples, where positive correlations are indicated in red and negative correlations in green

Heatmap analysis of IS datasets (GSE22255, GSE58294) revealed 279 differentially expressed genes (DEGs), with upregulated and downregulated genes represented in red and green, respectively (Fig. 2A-B). Weighted gene co-expression network analysis (WGCNA) identified an optimal soft threshold power of 7 for the IS cohort (Fig. 2C). Hierarchical clustering with dynamic tree cutting delineated 22 distinct gene modules (Fig. 2D). The MEcyan module demonstrated the strongest negative correlation with IS phenotypes (*r* = -0.63, *P* =3e^− 16^) (Fig. 2E). Scatterplot analysis confirmed a significant positive correlation between module membership and gene significance for MEcyan (cor = 0.81, *P* = 7.5 e^− 26^), validating the module’s association with disease pathogenesis (Fig. 2F).

### Enrichment analysis and PPI network construction of shared driver genes between IS and aging

**Fig. 3:**
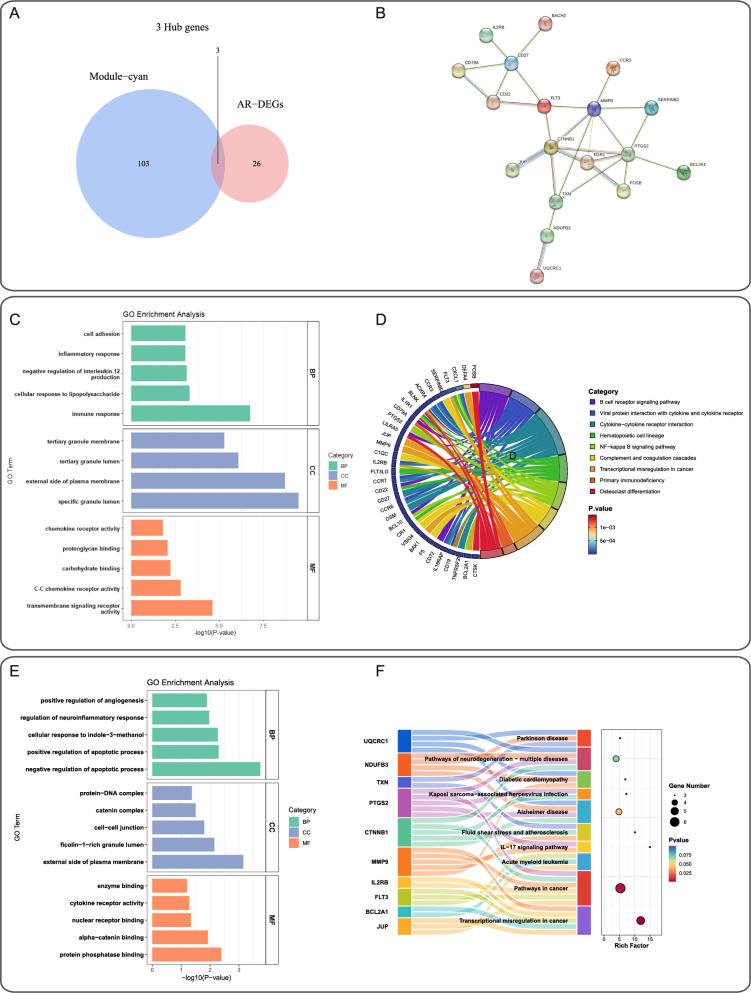
Functional and pathway enrichment analysis of shared driver genes between ischemic stroke (IS) and aging-related genes (ARGs). ​**A** Venn diagram showing the intersection of DEGs and ARGs. ​**B** Protein-protein interaction (PPI) network analysis of DE-ARGs. **C**, **D** Gene Ontology (GO) and Kyoto Encyclopedia of Genes and Genomes (KEGG) analyses elucidating the biological characteristics of DEGs. **E**, **F** GO and KEGG analyses exploring the biological features of DE-ARGs

GO analysis of 279 DEGs revealed significant enrichment in biological processes including immune response, inflammatory response, and cell adhesion (Fig. 3C). Cellular component analysis localized these genes to granular structures and membrane compartments, while molecular function analysis highlighted receptor-mediated signaling and binding activities. KEGG pathway analysis identified crucial pathways: B-cell receptor signaling, cytokine-cytokine receptor interaction, *NF-κB* signaling, hematopoietic cell lineage, complement and coagulation cascades, transcriptional dysregulation in cancer, primary immunodeficiency, and osteoclast differentiation (Fig. 3D).

Intersection analysis identified 29 aging-related DEGs (ARDEGs) (Fig. 3A). PPI network construction demonstrated robust protein interactions among these genes (Fig. 3B). GO enrichment of ARDEGs showed involvement in “positive regulation of angiogenesis,” “regulation of neuroinflammatory response,” and “cellular response to indole-3-carbinol.” KEGG analysis revealed enrichment in neurodegenerative diseases (Parkinson’s and Alzheimer’s diseases) and cancer-related pathways (Fig. 3E-F).

### Identification and validation of potential shared hub genes using SVM and LASSO

**Fig. 4:**
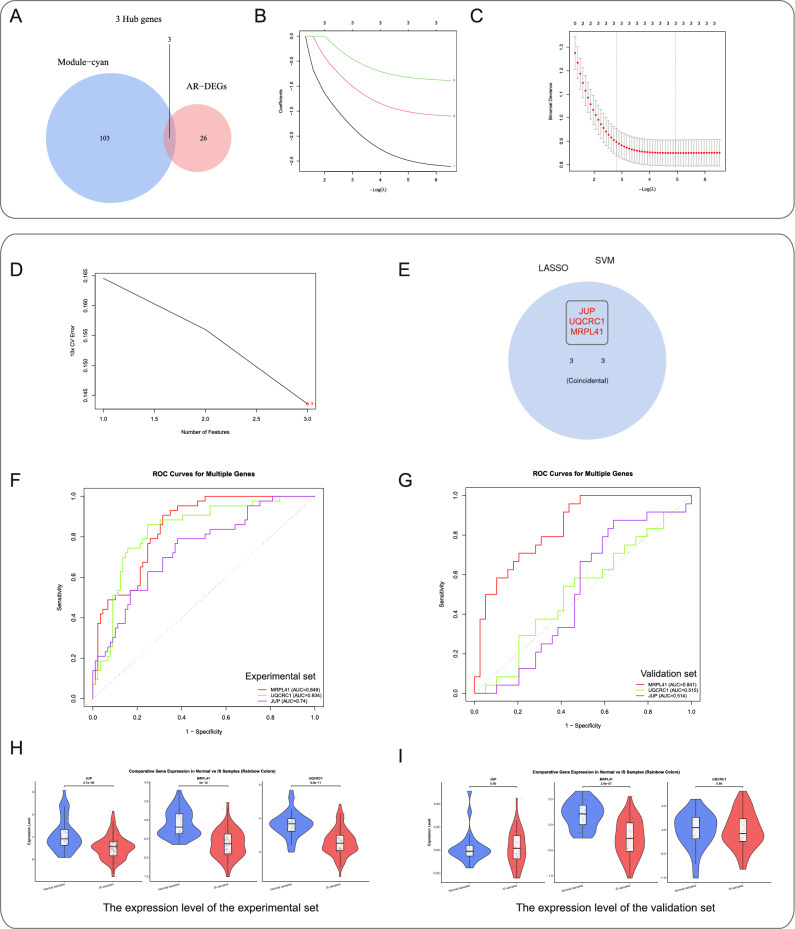
Screening of shared driver genes between IS and ARGs using LASSO and SVM algorithms. **A** Venn diagram showing the intersection of Module-cyan and AR-DEGs. **B**-**D **In-depth analysis of IS cohort data via LASSO regression and SVM algorithm . ​**E** Cross-validation integrating SVM and LASSO algorithms to identify the most representative hub gene set.​ **F**-**I** ROC curves of three shared potential diagnostic biomarkers and validation of their expression levels in both experimental and validation cohorts

Integration of ARDEGs with module genes yielded 3 candidate hub genes (Fig. 4A). LASSO regression analysis identified three potential biomarkers (Fig. 4B-C), while SVM algorithm optimization confirmed optimal diagnostic accuracy with these three genes (Fig. 4D). Cross-validation established *JUP*,* UQCRC1*, and *MRPL41* as robust diagnostic markers (Fig. 4E). ROC curve analysis demonstrated high diagnostic efficacy in experimental set (AUC: *MRPL41* = 0.848, *UQCRC1* = 0.834, *JUP* = 0.74; all > 0.7) (Fig. 4I). In addition, we conducted an ROC curve analysis on the external validation set (AUC: *MRPL41* = 0.841, *UQCRC1* = 0.515, *JUP* = 0.514; all > 0.5). Boxplot analysis confirmed significant downregulation of all three markers in IS patients (Fig. 4H), with *MRPL41* and *UQCRC1* showing consistent differential expression in validation cohorts (Fig. 4I).

### Immune cell infiltration and correlation with shared core genes

**Fig. 5:**
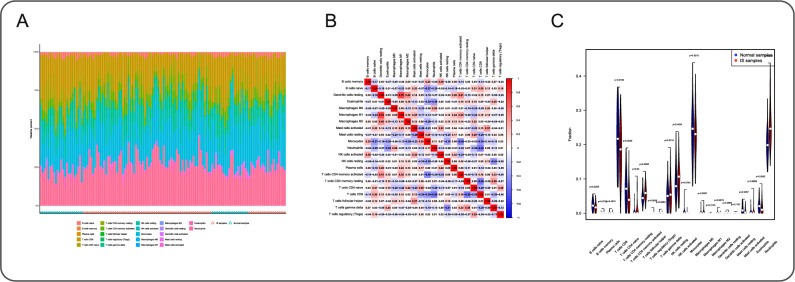
Immune cell infiltration analysis. **A** Relative proportions of 22 immune cell types. **B** Correlation matrix of all 22 immune cell subtypes, where higher, lower, and similar correlation levels are represented in red, blue, and white, respectively. ​**C** Boxplot illustrating immune cell infiltration patterns in the IS cohort, with blue indicating healthy controls and red indicating IS patients

CIBERSORT analysis revealed distinct immune cell proportions between IS patients and controls (Fig. 5A). The correlation heatmap demonstrated strong positive association between Macrophages M1 and Dendritic cells resting (*r* = 0.7), and negative correlation between T cells CD8 and T cells CD4 memory resting (*r*=-0.5) in IS patients (Fig. 5B). Differential infiltration analysis showed significant increases in T cells CD4 memory resting/activated, Macrophages M0, and Neutrophils, with decreases in B cells naive, T cells CD8, Mast cells resting, and Eosinophils in IS samples (Fig. 5C).

### Single-cell resolution analysis of core genes using DISCO platform

**Fig. 6:**
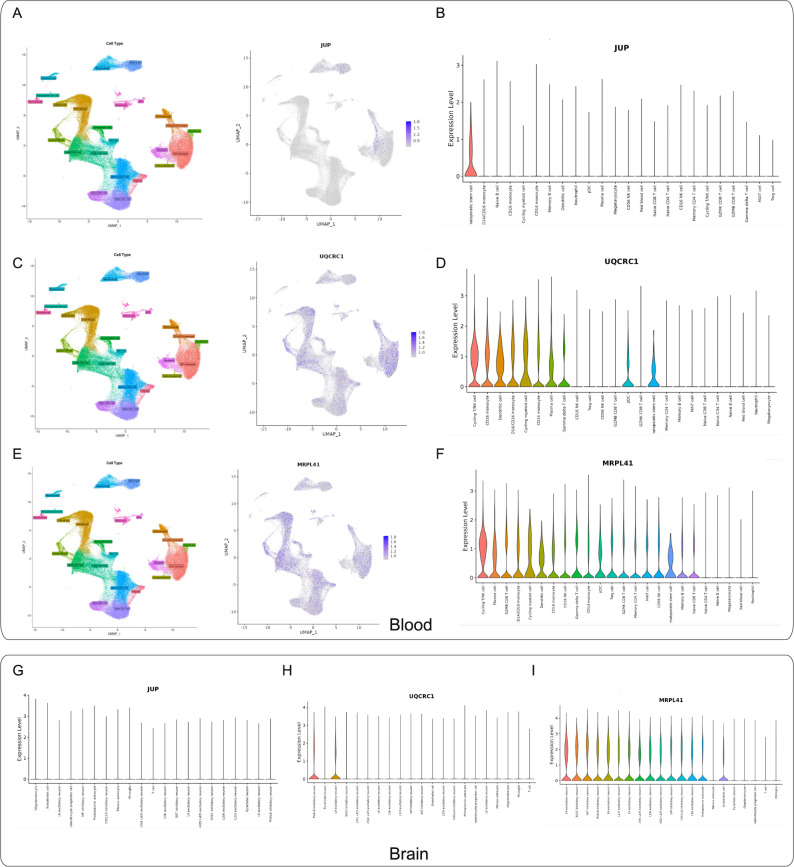
Single-cell analysis of hub gene expression profiles. **A**,**C**,**E** UMAP visualization of core genes across various blood cell types. ​**B**,**D**,**F** Expression levels of JUP, UQCRC1, and MRPL41 in different blood cell types. ​**G**-**I** Violin plots demonstrating the expression patterns of JUP, UQCRC1, and MRPL41 across distinct brain cell populations

UMAP visualization revealed cell-type specific expression patterns of hub genes (Fig. 6A, C and E). *JUP* showed predominant expression in hematopoietic stem cells with minimal brain expression (Fig. 6B and G). *UQCRC1* was highly expressed in blood immune cells (Cycling T/NK cells, CD14/CD16 monocytes) and brain neurons (PVALB inhibitory and L4 excitatory neurons) (Fig. 6D and H). *MRPL41* exhibited broad expression in blood immune cells and brain neurons/endothelial cells, with low expression in oligodendrocytes and microglia (Fig. 6F and I).

### Identification of candidate miRNAs and therapeutic compounds

**Fig. 7:**
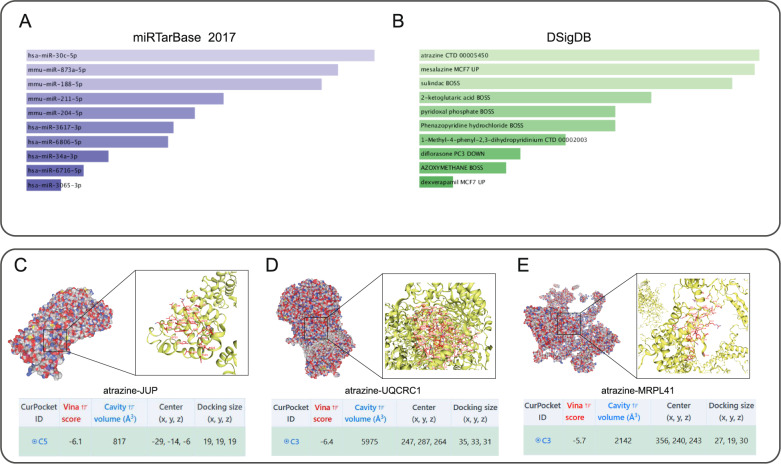
**A** Top 10 significant miRNA candidates targeting hub genes for IS-related aging therapy. **B** Top 10 significant drug candidates targeting hub genes for IS-related aging therapy. **C**-**E** Molecular docking diagram of atrazine with IS-related aging genes

miRTarBase 2017 analysis identified top candidate miRNAs targeting hub genes (q = 0.0218), including hsa-miR-30c-5p (predicted to regulate *JUP/UQCRC1*) and others targeting *MRPL41* (Fig. 7A; Table 2). DSigDB screening revealed potential therapeutic compounds (q = 0.0496), with atrazine showing the strongest predicted interactions (Fig. 7B; Table 3). Molecular docking demonstrated stable binding of atrazine to *JUP* (-6.1 kcal/mol), *UQCRC1* (-6.4 kcal/mol), and *MRPL41* (-5.7 kcal/mol), with binding poses visualized (Fig. 7C-E). The high absolute binding energies (<-6 kcal/mol for *JUP/UQCRC1*) suggest strong potential for functional modulation of these targets.

**Table 2:** Top 10 miRNA-target interactions from MiRTarBase 2017 ranked by statistical significance

Term	*P*-value	Adjusted *P*-value	Combined Score	Genes
hsa-miR-30c-5p	0.001989	0.021829981	467.7982022	JUP; UQCRC1
mmu-miR-873a-5p	0.002548	0.021829981	3729.253306	MRPL41
mmu-miR-188-5p	0.002847	0.021829981	3252.888057	MRPL41
mmu-miR-211-5p	0.00554	0.026543518	1440.455342	MRPL41
mmu-miR-204-5p	0.006735	0.026543518	1133.789719	MRPL41
hsa-miR-3617-3p	0.00778	0.026543518	949.624009	MRPL41
hsa-miR-6806-5p	0.008078	0.026543518	906.6154367	MRPL41
hsa-miR-34a-3p	0.012101	0.034791373	549.514823	MRPL41
hsa-miR-6716-5p	0.014332	0.036625144	444.6829485	MRPL41
hsa-miR-3065-3p	0.016707	0.03741256	366.5419846	MRPL41

The Combine Score is a calculated composite metric that quantifies the strength and diversity of recorded evidence for a specific miRNA-target pair in the database. A higher score indicates stronger and more reliable supporting evidence for the interaction

**Table 3:** Top 10 most significant drug-target associations from DSigDB enrichment analysis

Term	*P*-value	Adjusted *P*-value	Combined Score	Genes
atrazine CTD 00005450	0.003265284	0.049667913	292494.3782	MRPL41; JUP; UQCRC1
mesalazine MCF7 UP	0.003296484	0.049667913	2718.11468	MRPL41
sulindac BOSS	0.003446153	0.049667913	2574.276639	JUP
2-ketoglutaric acid BOSS	0.004044678	0.049667913	2116.293582	JUP
pyridoxal phosphate BOSS	0.004343851	0.049667913	1939.487001	JUP
Phenazopyridine hydrochloride BOSS	0.004343851	0.049667913	1939.487001	JUP
1-Methyl-4-phenyl-2,3-dihydropyridinium CTD 00002003	0.004792498	0.049667913	1719.878826	UQCRC1
diflorasone PC3 DOWN	0.00524101	0.049667913	1541.625367	JUP
AZOXYMETHANE BOSS	0.005390484	0.049667913	1489.484609	JUP
dexverapamil MCF7 UP	0.005988231	0.049667913	1309.541399	MRPL41

The Combine Score is a calculated composite metric that quantifies the strength and diversity of recorded evidence for a specific miRNA-target pair in the database. A higher score indicates stronger and more reliable supporting evidence for the interaction

## Discussion

Ischemic stroke (IS) is a multifactorial disorder characterized by vascular dysfunction [16], inflammatory responses [17, 18], oxidative stress [19, 20], and neuronal injury [21]. While existing studies have partially elucidated its molecular mechanisms, the shared pathophysiology between IS and aging remains incompletely understood [22, 23].

In this study, we employed an integrative approach combining transcriptomics, weighted gene co-expression network analysis (WGCNA), machine learning, and immune infiltration profiling to systematically identify core driver genes shared by IS and aging, while evaluating their diagnostic and therapeutic potential.​.

### Biological significance and diagnostic potential of core genes

Through differential expression analysis and WGCNA, this study identified 279 IS-related DEGs, with 29 overlapping aging-related genes (ARDEGs). Among these, *JUP*, *UQCRC1*, and *MRPL41* emerged as the most significant hub genes. ROC analysis demonstrated significant downregulation of these genes in IS patients, with AUC values > 0.7, indicating high diagnostic accuracy. Single-cell analysis further validated their cell-type-specific expression patterns in neurons and immune cells, suggesting their potential as IS biomarkers.

Mitochondrial Ribosomal Protein L41 (*MRPL41*) plays a critical role in mitochondrial protein synthesis [24]. Its significant downregulation in IS patients’ blood correlates with immune cell infiltration and exhibits strong diagnostic potential (AUC > 0.8), implicating mitochondrial dysfunction in neuronal energy metabolism impairment and diminished neuroprotection post-stroke [25]. Mendelian randomization studies have also identified *MRPL41* overexpression as a risk factor for Alzheimer’s disease (AD). In cancer, *MRPL41* acts as a tumor suppressor by stabilizing p53 and promoting apoptosis, with low expression observed in renal and lung cancers [26].

Junction Plakoglobin (*JUP*), a member of the catenin family, is essential for cortical folding and lamination. While its role in other diseases is established, IS-specific mechanisms remain unclear. In skin aging, *JUP* mRNA and protein levels decline by 68–79% in aged individuals, compromising barrier integrity [27]. Cardiovascular studies reveal that low *JUP* expression in acute myocardial infarction predicts major adverse cardiovascular events (MACE; HR = 0.234, *P* < 0.05), supported by ROC analysis (AUC = 0.8299) [28]. In primates, *JUP* is widely expressed in prefrontal cortex and hippocampal neurons, with post-stroke downregulation potentially disrupting cell-cell signaling and tissue repair [29]. Our findings suggest *JUP*’s involvement in IS-aging comorbidity via epithelial barrier dysfunction, inflammation, or tissue remodeling, warranting further investigation into it IS-specific regulatory networks.

Ubiquinol-Cytochrome c Reductase Core Protein 1 (*UQCRC1*), a core component of mitochondrial Complex III, is pivotal in aging-related pathologies. In cumulus cells of infertile women (40–45 years), *UQCRC1* downregulation (*P* < 0.05) correlates with oxidative phosphorylation defects, impairing oocyte developmental competence [30]. Intriguingly, *UQCRC1* is a shared hub gene in AD and musculoskeletal aging, with tissue-specific dysregulation (downregulated in muscle but upregulated in AD) linked to neuronal bioenergetic failure [31]. This study highlights *UQCRC1* as a novel IS-aging hub gene, where its dysregulation may exacerbate mitochondrial dysfunction and oxidative stress, contributing to neuronal injury and vascular aging. Its diagnostic value (AUC = 0.834) and potential as a therapeutic target (e.g., taurine supplementation) warrant further exploration.

### Shared molecular foundations of aging biology and ischemic stroke

This study identified *JUP*,* UQCRC1*, and *MRPL41* as key genes shared between aging and ischemic stroke, with their expression significantly downregulated post-stroke. This finding is closely associated with several core mechanisms of aging, offering a new perspective for research into the mechanisms of aging-related cerebrovascular diseases.

Junctional plakoglobin (*JUP*) is a critical cell adhesion molecule involved in mediating intercellular adhesion. Kamil Oender et al. found that *JUP* expression is decreased in aged human skin, and *JUP* deficiency leads to *NLRP1* inflammasome activation, manifested by increased levels of *IL-1β* and *IL-18* [32]. *UQCRC1* is a subunit of mitochondrial complex III and plays a significant role in mitochondrial metabolism [33, 34]. Decreased expression of *UQCRC1* contributes to musculoskeletal aging (sarcopenia) by disrupting the mitochondrial microenvironment, and it can serve as a biomarker for musculoskeletal aging [31]. In vitro evidence indicates that overexpression of *UQCRC1* leads to increased phosphorylation of the *PI3K/Akt* signaling pathway and concurrently reduces apoptosis by decreasing caspase-3 activation [35]. Furthermore, studies have shown that *UQCRC1* dysfunction is associated with reduced mitochondrial complex III respiratory chain activity and diminished cerebral mitochondrial content [34], leading to disruption of cerebral mitochondrial bioenergetics [36, 37]. As we found, the significant downregulation of *UQCRC1* in IS patients may lead to decreased mitochondrial membrane potential and ATP content in the ischemic cerebral cortex [38], along with increased free radicals, thereby contributing to more severe neurological dysfunction in IS patients following cerebral ischemia/hypoxia or focal cerebral ischemia. *MRPL41* is a crucial regulatory node in the *p53* pathway, enhancing *p53* stability and mediating apoptosis. Concurrently, downregulation of *MRPL41* may impair mitochondrial function, leading to compromised neuronal energy metabolism and reduced neuroprotection. This aligns with the “mitochondrial theory of aging” – that mitochondrial dysfunction is increasingly recognized as a unifying mechanism underlying aging and various age-related diseases [39].

Existing research has not integrated the association of *JUP*, *UQCRC1*, and *MRPL41* with the “aging-ischemic stroke” axis. This study reveals molecular-level overlap between aging and ischemic stroke, suggesting the potential existence of shared mechanisms, which warrants further experimental validation in future research.

### Immune microenvironment dysregulation in aging-ischemic stroke comorbidity

Immune-inflammatory responses play pivotal roles in both ischemic stroke (IS) and aging. Our immune infiltration analysis revealed significant increases in M1 macrophages, neutrophils, and memory T cells in IS patients, alongside reduced CD8⁺ T cells and resting mast cells, consistent with a pro-inflammatory microenvironment.

M1 Macrophage Polarization: Post-stroke microglia/macrophages shift from an early anti-inflammatory state to a pro-inflammatory M1 phenotype, driven by *STING*-mediated type I interferon signaling. Inhibiting *STING* blocks this transition, reduces neuroinflammation, and improves functional recovery [40]. Aging exacerbates this process via vitamin D deficiency, which impairs vitamin D receptor (*VDR*) signaling in macrophages, augmenting *TNF-α/IFN-γ* release and M1 polarization [41].*NF-κB* and Cytokine Receptor Pathways: *NF-κB* activation amplifies pro-inflammatory cytokine release (e.g., *TNF-α*,* IFN-γ*), disrupting blood-brain barrier (BBB) integrity and recruiting peripheral T cells. *FGF21* ameliorates neuroinflammation by suppressing *NF-κB* while activating *PPAR-γ*, reducing M1 polarization and neutrophil infiltration [42]. Central-Associated Macrophages (CAMs) in Aging-IS Link: Infiltrating monocyte-derived macrophages (CAMs) protect BBB integrity via *IL-33/ST2* signaling. ST2 deficiency or macrophage depletion abolishes this protection, increasing infarct severity [43]. Aging impairs CAM function through *VDR* loss, promoting pro-inflammatory phenotypes. Shared Mechanisms with Neurodegenerative Diseases: Post-stroke dysregulated phagocytosis (e.g., via *MEGF10/MERTK*) causes synapse loss, mimicking neurodegeneration patterns [44]. *METTL14*-mediated m⁶A modification upregulates *KAT3B*, enhancing *STING* promoter acetylation and *NLRP3* inflammasome activation—a mechanism also implicated in Alzheimer’s disease [45]. The “immune-aging axis” exacerbates IS through *STING-NF-κB*-driven inflammation, CAM dysfunction, and impaired phagocytosis/mitochondrial pathways. Targeting *STING*, *VDR*,* or IL-33/ST2* may mitigate age-related stroke vulnerability.

### Identification of potential therapeutic targets and drug prediction

Through in silico molecular docking and database screening, we identified candidate compounds and miRNAs potentially capable of modulating the core genes. Computational analysis suggested that atrazine, a triazine herbicide, exhibits binding affinities to *JUP*,* UQCRC1*, and *MRPL41*, with binding energies ranging from − 5.7 to -6.4 kcal/mol. However, it is critical to emphasize that atrazine is an environmental toxicant with recognized neurotoxicity and is banned in numerous countries.​ Thus, its identification here serves solely to indicate the potential “druggability” of these target proteins—not to suggest atrazine itself as a viable therapeutic candidate. The moderate binding energies observed, while indicating potential interaction, are considerably weaker than the high-affinity thresholds typically considered biologically significant (often < -8 kcal/mol). Furthermore, these preliminary computational results do not account for critical pharmacological parameters such as blood-brain barrier penetration, metabolic stability, or off-target effects.

Similarly, hsa-miR-30c-5p was computationally predicted to target *JUP* and *UQCRC1*, suggesting a potential role in post-stroke regulation of cellular junctions and mitochondrial function. Drug repositioning analysis also highlighted candidates like *mesalazine*, which may influence inflammatory pathways relevant to IS. It is imperative to state unequivocally that all these predictions—for *atrazine*, miRNAs, and repositioned drugs—are hypothesis-generating and derived purely from database mining and docking simulations. They lack experimental validation and their biological relevance in the context of ischemic stroke remains entirely speculative.

Therefore, the primary value of this analysis lies in proposing novel regulatory nodes and protein targets for future investigation. Rigorous in vitroand in vivostudies are essential next steps to evaluate the true therapeutic potential of modulating these targets with specific, safe, and brain-penetrant molecules..

### Study limitations and future Directions

While this study elucidates molecular links between IS and aging, limitations include:

Independent cohorts are needed to validate the robustness of *JUP*, *UQCRC1*, and *MRPL41* as biomarkers; Genetic knockout/overexpression experiments are required to confirm the functional roles of these genes in IS pathogenesis; Single-cell spatial transcriptomics could resolve dynamic immune cell changes during IS progression. The clinical feasibility of *JUP*, *UQCRC1*, and *MRPL41* as potential biomarkers for prognostic prediction in ischemic stroke (IS) requires further evaluation. Studies indicate that *JUP* reflects cellular junction stability, and its downregulation may disrupt intercellular connections, potentially influencing apoptosis via the *Wnt/β-catenin* signaling pathway [46]. Our findings confirm the high diagnostic efficacy of *JUP* (AUC = 0.74), with cross-validation identifying it as a reliable diagnostic marker. Its expression levels may correlate with post-IS outcomes, suggesting that *JUP* holds advantages in assessing long-term prognosis in IS patients by directly reflecting alterations in cellular junctions and structure. Monitoring *JUP* levels in IS patients could facilitate early identification of high-risk individuals, thereby informing personalized therapeutic strategies. Zhang et al. demonstrated that *UQCRC1* deficiency leads to defects in the mitochondrial respiratory chain [47], resulting in lipofuscin accumulation and increased neuronal apoptosis [48]. Conversely, *UQCRC1* overexpression has been shown to protect cells against ischemia-reperfusion injury [35], implicating its dysregulation in adverse IS prognoses. *MRPL41* plays a role in mitochondrial protein synthesis and has been linked to stroke pathophysiology [49]. In IS patients, downregulation of *MRPL41* may impair mitochondrial function, leading to compromised neuronal energy metabolism and reduced neuroprotection, potentially affecting post-stroke recovery [50]. The potential correlation of *JUP*, *UQCRC1*, and *MRPL41* with IS severity and prognosis further underscores the clinical relevance of these genes. While this study is primarily based on GEO database analyses, subsequent validation using independent clinical samples is essential. Specifically, future multi-center clinical trials with larger cohorts should systematically analyze the association between clinicopathological features and adverse outcomes in IS patients, while examining expression changes of *JUP*, *UQCRC1*, and *MRPL41* in blood and brain tissues. The validation approach should include: Comparative analysis of clinical data: Correlate clinicopathological characteristics with adverse outcome indicators, such as changes in NIHSS and mRS scores, infarct volume, serious adverse events within 90 days (including prolonged hospitalization, permanent disability, life-threatening events, or death), vascular events (e.g., recurrent symptomatic ischemic stroke, myocardial infarction, or vascular mortality), and the proportion of patients with favorable functional outcomes [51].Expression profiling: Compare *JUP*, *UQCRC1*, and *MRPL41* expression levels in blood and brain tissues between groups with and without adverse events. Predictive value assessment: Employ ROC curve analysis to evaluate the prognostic predictive value of each marker for adverse outcomes in IS patients.

Future studies should integrate organoid models, multi-omics data (e.g., methylation + proteomics), and AI-driven prediction to optimize personalized IS therapies, particularly for elderly patients with immunosenescence.

## Conclusion

This study systematically reveals shared molecular mechanisms between IS and aging, identifying *JUP*, *UQCRC1*, and *MRPL41* as potential diagnostic biomarkers and therapeutic targets. Immune microenvironment dysregulation, particularly macrophage polarization and T-cell subset alterations, may bridge aging and IS pathology. The computationally predicted interactions with *atrazine* and *hsa-miR-30c-5p* highlight potential regulatory nodes​ that warrant further experimental investigation to assess their true therapeutic relevance. Further exploration of their clinical translatability is critical to improving outcomes in aged IS populations.

## Supplementary Information

Supplementary Material 1.

## Data Availability

The data underlying the results of this study can be obtained from the corresponding author when a reasonable request is made.
